# Fixed Full-Arch Implant-Supported Restorations: Techniques Review and Proposal for Improvement

**DOI:** 10.3390/dj12120408

**Published:** 2024-12-13

**Authors:** Florin-Octavian Froimovici, Cristian Corneliu Butnărașu, Marco Montanari, Mihai Săndulescu

**Affiliations:** 1 Doctoral School, “Carol Davila” University of Medicine and Pharmacy, 050474 Bucharest, Romania; 2 MINEC—MegaGen International Network of Education & Clinical Research, 030925 Bucharest, Romania; 3 Department of Prosthodontics, University of Modena e Reggio and Ferrara, 44121 Ferrara, Italy; 4 Department of Implant-Prosthetic Therapy, Faculty of Dentistry, “Carol Davila” University of Medicine and Pharmacy, 050474 Bucharest, Romania; mihai.sandulescu@umfcd.ro

**Keywords:** fixed full-arch zirconia, hybrid restoration, titanium implant bar

## Abstract

Full-arch zirconia restorations on implants have gained popularity due to zirconia’s strength and aesthetics, yet they are still associated with challenges like structural fractures, peri-implant complications, and design misfits. Advances in CAD/CAM and digital workflows offer potential improvements, but a technique that consistently addresses these issues in fixed, full-arch, implant-supported prostheses is needed. This novel technique integrates a facially and prosthetically driven treatment approach, which is divided into three phases: data acquisition, restoration design, and manufacturing/delivery. Digital tools, including intraoral scanning and photogrammetry, facilitate accurate implant positioning, while 3D design software enables functional and aesthetic validation before final milling. A dual software approach is used to reverse engineer a titanium bar from the final restoration design, ensuring a superior outcome to other protocols. The restoration incorporates a zirconia–titanium hybrid structure, optimizing strength, flexibility, and weight. The proposed workflow enhances restoration precision and predictability through a prosthetically driven treatment plan, by ensuring passivity and aligning with biological and mechanical principles to promote long-term stability. By starting with the proposed restoration design and reverse engineering the bar, while also allowing for flexibility in material and component choices, this technique accommodates both patient needs and financial considerations. This approach demonstrates potential for improving patient outcomes in full-arch implant restorations by minimizing complications associated with traditional methods. Further research is recommended to validate the technique’s efficacy and broaden its clinical applications.

## 1. Introduction

Edentulism, though extensively discussed in the literature, significantly impacts patients, leading to impaired masticatory function, speech, aesthetics, and a decline in overall quality of life [1]. It is considered that the incidence of edentulism may rise with increasing life expectancy, yet improved quality of life, particularly in developed nations, may reduce the rate. Nonetheless, the World Health Organization reports a prevalence of edentulism ranging from 1.24% to 16.85% among individuals older than 20 years of age [2]. Given the profound functional and aesthetic consequences, which can precipitate behavioral and psychological changes [3], it is of no surprise that in the past few years, surgical and prosthetic workflows increasingly prioritize providing an implant-supported fixed type of restoration.

A study published in 2019 involving 116 prosthodontists from 33 countries revealed considerable variety when providing a similar treatment option, a mandibular full-arch implant-supported restoration [4]. While the study indicated a preference among prosthodontists for implant retained overdentures, the majority reported the application of 1 to 10 fixed appliances throughout their patients in 2015, with materials employed including milled titanium frameworks and acrylic or porcelain teeth, milled chrome–cobalt and acrylic teeth or ceramic veneers, monolithic zirconia and fiber-reinforced composite frameworks [4].

Despite this diversity, the common denominator remains the use of three or more dental implants, with extensive support for the all-on-four treatment concept [5,6,7]. Conversely, overdentures with attachments only require the placement of one or two dental implants [8]. When conditions are met for the placement of three or more implants, this infrastructure proves adequate for both removable and fixed prosthodontics. These solutions provide numerous benefits for the patient, although they differ significantly in various aspects, including stress distribution, passive fit, bone loss over time, comfort, esthetic outcome or hygiene requirements [9,10,11]. Studies evaluating patient satisfaction often focus on the oral health index profile, comfort, stability, chewing efficiency, speaking ability, esthetics, presence of pain during wear and cleaning. Consequently, the preference for fixed prosthodontics is understandable [10,11]. Moreover, while fixed prosthodontics may be desirable, any enhancement in retention and stability over the conventional denture is advantageous, as it improves muscle activity and overall masticatory function [12]. ELsyad et al. (2020) shows that both fixed prostheses and milled bar overdentures increase the amplitude and time of the masticatory cycle while reducing the chewing rate and masticatory time, compared to conventional dentures. The authors discuss the potential ossteoperception of the underlying implants and the number of occluding teeth for implant-supported restorations and the displacement accompanied by discomfort and pain of the conventional denture [12].

## 2. Aim and Rationale

The aim of this paper is to show the abundance of options a clinician faces when providing a fixed full-arch implant-supported restoration and to present a new prosthetic concept, building on the materials and technology available today. Implant-supported full-arch restorations must exhibit satisfactory mechanical properties, traditionally relying on high-strength materials for the entire structure or, at the very least, for the framework. The spectrum of framework materials is wide, encompassing gold alloys, titanium, silver–palladium alloys, zirconia and polymers such as acrylic resins, fiber-reinforced composites and polyetheretherketone (PEEK) [13,14]. Recent studies have also explored graphene as a potential framework material, although literature on this remains limited [15]. For the scope of this paper, our focus will center on the applications of titanium as a bar and framework material.

Traditional titanium frameworks, fabricated through laser welding of prefabricated components, have been utilized for over three decades [16]. These structures have demonstrated favorable properties, with multiple studies proving their comparability to gold castings over many years [17,18,19]. More recently, intraoral welding has been utilized for the immediate loading of dental implants, yielding success for both fixed and removable prostheses [20,21,22,23,24]. The same protocol can be used with little to no adaptation for augmented sites or implants placed in challenging anatomical situations [25,26].

With the advent of digitalization in dentistry, traditional methods have given way to modern techniques such as computer numerical control (CNC) milling and 3D printing. These advancements have markedly enhanced the planning, execution and outcomes of treatment. CNC milling, in particular, offers superior fit and accuracy for implant prosthodontic frameworks compared to casting or welding [27,28]. This explains the prevalence of computer-aided design/computer-aided manufacturing (CAD/CAM)-enhanced workflows in contemporary protocols.

The proposed treatment approach involving a zirconia superstructure cemented over a titanium bar presents an alternative to other procedures documented in the literature. The fabrication of the bar involves a reverse engineering process utilizing dental design software, starting from the ideal restoration.

## 3. Treatment Concepts

While fixed dentures and full-arch zirconia restorations often utilize monolithic designs, the majority of long-term fixed full-arch restorations incorporate at least two materials to achieve the desired functional and aesthetic properties. Additionally, advancements in technology and the evolving role of the dental technician into a dental designer are evident in the diversity of techniques employed. These range from traditional analog methods—such as the Ohio State University technique, Brånemark Novum protocol, Trefoil System, and Stumpel substructure—to modern digitally planned approaches that utilize 3D-printed or milled components, including Malo/Toronto bridges, bars with superstructures, and full-arch zirconia restorations.

The following section provides a review of various treatment options for a similar clinical scenario, emphasizing that the choice often relies on the skill and experience of the clinician, with no definitive indication favoring one over another.

### 3.1. The Fixed Denture

Primarily employed in cases necessitating a full denture with adequate underlying bone support, implant fixed complete dentures significantly enhance patients’ quality of life, encompassing improvements in aesthetics, phonetics and function. Extensively utilized for many years, long-term studies have conclusively demonstrated its superiority over traditional dentures in terms of patient satisfaction [29]. With or without a framework, material costs are economical, and even with just three implants, satisfactory outcomes can be achieved with minimal complications. This method is reported with a success rate exceeding 95% for implant survival and 97% for prosthetic survival during a 1–6-year follow-up, with common complications including screw loosening and acrylic component fracture [30]. Although catastrophic failures may be avoided, certain wear over time is well-documented, specifically depending on the opposing dentition. Moreover, there are studies that consider the fixed acrylic denture’s severe wear over time as a failure [31,32]. Even so, a notable advantage provided by this approach is the capacity to adapt for fast and cost-effective rehabilitation through immediate loading, thereby shortening treatment duration [6].

### 3.2. The Ohio State University Method

Utilizing the Ohio State University acrylic frame, this method emphasizes adaptability and expedites the production of the final prosthetic framework. The protocol reports spanning 4 days and involves the intraoral assembly of a prefabricated acrylic bar, waxing sleeves and autopolymerizing acrylic resin to achieve passivity between implants. Subsequently, this acrylic ensemble is cast into a metal framework supporting acrylic teeth and gingiva [33]. Although it appears to improve the reliability of the registration of implant placement, this technique may not align with modern, digital workflows due to its labor-intensive nature, posing challenges for both clinicians and dental technicians.

### 3.3. Brånemark Novum Protocol

This well-documented protocol, in use for over 50 years, has evolved from Co-Cr cast frameworks to precious alloys and more recently to milled titanium. Standardized guided drilling templates are used for the insertion of the implants, with a 3- to 6-month timeframe typically allocated for osteointegration, but immediate loading can be a better alternative for patient comfort. The protocol commonly involves implants placed in the anterior mandible using a surgical guide, connected via a prefabricated titanium bar, also called the primary bar. The secondary bar forms the prosthetic superstructure, screwing into the primary bar and supporting an acrylic structure with conventional denture teeth. Despite its success, the protocol entails many components in both the surgical and prosthetic phases. The advantages claimed by this technique include the rigid splinting of the implants, the availability of prefabricated components that require little to no adaptation in order to achieve fit and passivity and a quicker treatment time compared to other methods available at the time [34,35]. However, modern approaches allow for pre-surgical planning and immediate loading with a provisional, maximizing adaptability to patients’ bone and ideal prosthetic outcome.

### 3.4. Trefoil System

Consisting of a prefabricated titanium bar mountable on three implants, the system offers a certain degree of fixation adaptation through bar slots and implant connections, allowing for 0.4 mm horizontal and 0.5 mm vertical adjustments, with 4 degrees of inclination [36]. While somewhat similar to the Brånemark Novum protocol, the Trefoil’s system emphasizes adaptability through the prefabricated bar. Nonetheless, though prefabricated components reduce treatment time and provide predictability, the reduced costs and great precision of CAD/CAM manufacturing allow for quick and reliable protocols that can adapt to the patient better than any prefabricated system.

### 3.5. Stumpel Modular Substructure

Past experiences with fixed acrylic dentures have revealed that achieving successful fixed full-arch prosthodontics demands a more rigid framework than acrylic resin, as complications often arise from a lack of adaptation of the prosthetic component rather than issues at the bone-implant interface, though implant failure remains a possibility in severe cases [37,38]. L.J. Stumpel introduced a novel technique in 2020 that addressed these challenges by assembly of implant-supported modular components intraorally and luting with composite resin. The system offers adaptability and passivity by luting bar components onto a laboratory cast with the implant cylinders directly on the implant analogs. Claiming to create a custom titanium framework in 45 min with same-day delivery, this system presents promising efficiency [39]. However, the involvement of multiple components and a labor-intensive process may pose challenges for inexperienced practitioners, while also necessitating further studies on mechanical behavior and potential complications associated with multiple interfaces within the framework structure.

### 3.6. The Malo/Toronto Bridges

The Malo or Toronto bridges consist of a metal framework that screws directly to implants or multiunit abutments, providing necessary support for cemented individual crowns to rehabilitate missing arches [40]. However, in our experience, shaping the bar to include abutments for the crowns poses certain challenges during manufacturing, particularly concerning the insertion axis for individual tooth restorations. Material options for the framework include titanium, Co-Cr alloy or glass fiber-reinforced resin [41], while acrylic resin or porcelain is used for the gingiva. Crowns can be fabricated from ceramics or from porcelain fused to metal [42]. Connection to implants can be through standard multiunit abutments or more recently, via the OT-bridge system (Rhein 83 S.R.L., Bologna, Italy) [41]. This restoration type has demonstrated good clinical outcomes and serves as a reliable alternative to bar retained overdentures [43].

### 3.7. The Bar with Sectioned Restorations

An alternative to the Malo or Toronto bridge, this method offers versatility and freedom in creating similar restorations. Utilizing a titanium bar, this technique supports restorations for multiple teeth in arch segments. The cited article describes the splitting of each arch into three segments and cementing metal–ceramic segments onto the titanium bar with acrylic gingiva [44]. This approach overcomes the challenge of multiple insertion axes for overlying crowns while reducing the risk of failure during crown cementation.

### 3.8. Complete Arch Zirconia Screwed to a Metal Bar

This protocol involves creating a metal Co-Cr bar with multiunit cylinders for a full arch on implants with significant inter-implant space. The metal structure provides high tensile strength and allows segmentation of the zirconia superstructure, due to size limitations of zirconia presintered discs for milling. The case shown by A.S. Bidra, 2020, achieves screw anchorage of the zirconia through tapped holes in the bar, creating two accessible interfaces between the arch and the implants: one between the bar and the implants and one between the zirconia and bar [45]. While this restoration type addresses some disadvantages of the full-arch zirconia restoration, it also introduces another potential point of failure and increases design complexity. Nevertheless, it offers clinicians and dental technicians the flexibility to design complex fixed full-arch dental prostheses tailored to individual cases.

### 3.9. Full-Arch Zirconia

Zirconia full-arch fixed restorations represent a functional and aesthetically pleasing treatment option for edentulous patients. Zirconia can be used either in a monolithic form, with or without cylinders for connection to multiunit abutments, or veneered to provide excellent esthetics for both teeth and gingiva. However, a recent review has shown that these restorations require further research with randomized clinical trials, as reported studies lack key aspects of standardization, such as the indications of each type of zirconia and other design elements when milling the restorations [46]. Despite their benefits, these restorations are associated with complications that could be avoided or better managed by expanding research or by exploring other types of structures. The issues taken into consideration for a fixed full-arch restoration usually include biologic complications derived from peri-implant bone loss, peri-implantitis or mucositis and technical challenges related to structure design and interfaces, such as fractures, component debonding, chipping, screw fracture or loosening, damage to antagonists or unpleasant sounds [47]. Another significant aspect reported was the structural fracture, most likely linked to the design of the restoration [48]. The high modulus of elasticity of zirconia may exacerbate misfit issues and affect underlying implants, although this problem could be somewhat mitigated by using intermediate abutments [49]. As fixed full-arch structures increasingly rely on virtual planning and milling or 3D printing technologies, collaborative planning between dental professionals and technicians is essential for optimizing treatment outcomes.

## 4. Proposal for Improvement

### 4.1. The Choice

The multitude of restorations described provides an option for every dental office, using different materials and technologies, each with its own learning curve. Therefore, while taking into account that not all clinicians are using digital technology, studies nowadays focus on the opportunities of digital workflows, with a clear shift towards the standard of having an intraoral scanner and learning digital protocols. As the clinician learns to incorporate the digital acquisition of information, so does the dental technician learn to use CAD software for designing restoration. The requirements of implementing a novel technique have increased through the use of more specialized digital devices that offer improvements over their first iterations. For the updated practitioner, the choice is therefore left between only a few types of restoration, although this does not clarify which is the best alternative. Toronto bridges, bars with sectioned superstructures, zirconia screwed to a bar or full-arch zirconia could all be treatment options for a similar case, all of which require similar digital expertise in order to achieve a satisfactory result.

### 4.2. The Proposed Novel Technique

The proposed technique builds on recent developments with CAD/CAM software and machining, by having a facially and prosthetically driven treatment plan. A somewhat similar technique is described by Pelekanos et al. in 2023, but uses a different workflow to achieve the desired result [50].

The protocol can be divided into three phases, starting after the case preparation for the prosthodontic stage: data acquisition, restoration design and restoration manufacturing and delivery. Data acquisition can be carried out from scratch or it can be carried out before other pre/proprosthetic interventions, depending on the partial or full digitalization of the patient. After obtaining all the required information, restoration design can commence. Depending on the reproducibility of the protocol, the relation between clinician and dental technician and level of confidence, manufacturing and validation can overlap with the design stage, allowing for multiple try-ins for each component. As a principle, ideal planning can be carried out before implant placement. However, as modifications may occur, data may be reacquired when starting the final restoration.

#### 4.2.1. Data Acquisition

**Figure 1:**
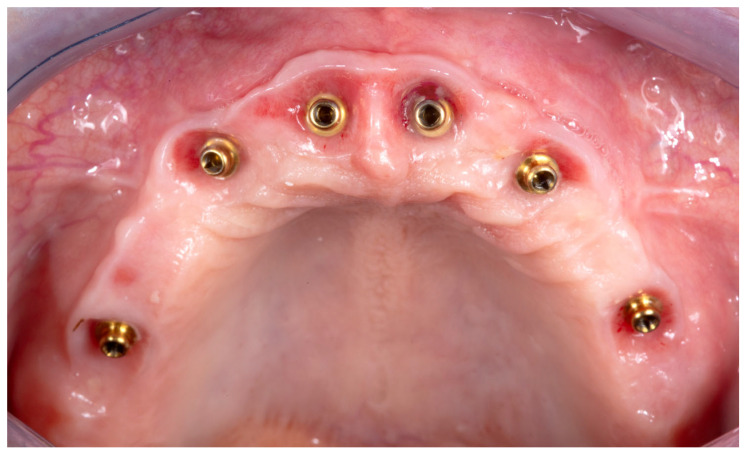
Prosthetic field before data acquisition.

Considering the patient is in the prosthetic phase of the treatment (Figure 1), after the implants were placed, with no other data acquired, the following information is needed:

1. Assessment of implant positioning
  - Optical registration with an intraoral scanner (IOS) or with a photogrammetry device must be carried out; photogrammetry has been shown to have better accuracy when superimposing implant positions for all-on-X-type restorations [51].
  - Conventional pick-up impression with splinted impression copings, followed by model casting with implant analogs and model scanning must be carried out directly in the dental office, or the technician can provide a jig to be splinted intraorally.
  - A mixed protocol may allow the use of an intraoral scan for gingiva and implant positioning, followed by a bonded jig for precision assessment, model casting and scanning and finally overlapping the first and second scans.
2. Registration of gingiva can be carried out at the same time as the implant positioning assessment or it can be scanned/impression-model-scanned and digitally overlapped.
3. Vertical dimension of occlusion (VDO) can be assessed using photography, facial scanning, radiographs or occlusal rims.
4. Intermaxillary occlusal relations may be assessed through either static or dynamic occlusal registration.
  - Static occlusion registration—through the use of occlusal rims.
  - Dynamic occlusion registration—through the use of devices that capture and record real jaw motion.

#### 4.2.2. Restoration Design and Validation

**Figure 2:**
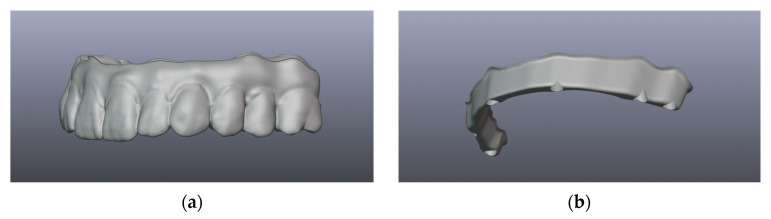

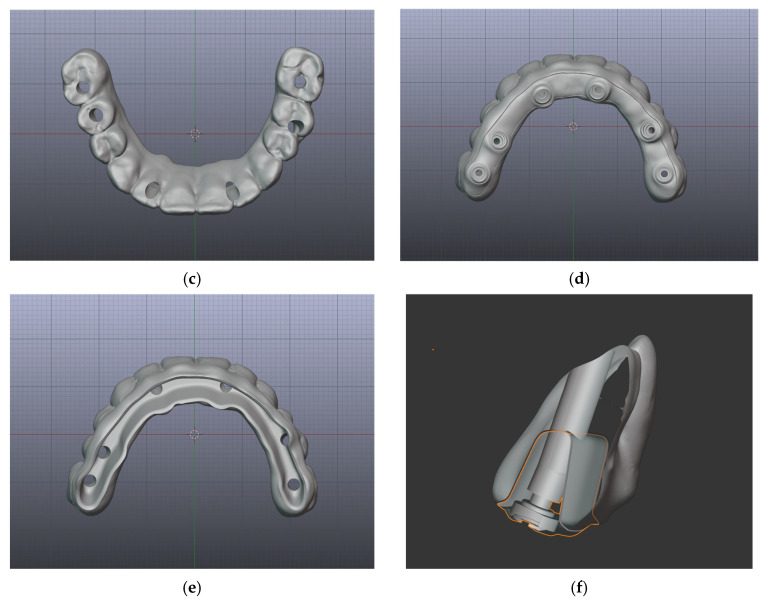
Example of bar and superstructure design. (**a**) Halfprofile view of the final restoration design imported into the Blenderfordental software. (**b**) View of the bar, sectioned from the final restoration. (**c**) Occlusal view of esthetic component, without the titanium bar, with screw-hole design. (**d**) Gingival view of entire restoration, bar and superstructure, after reverse engineering the bar—the overall restoration retains its shape and size. (**e**) Gingival view of the superstructure design, without the bar. (**f**) Sectioned view through a screw-hole, highlighting the bar design, showing the screw-hole design through bar and superstructure.

The design concept involves using Exocad (exocad GmbH, Darmstadt, Germany) for the final restoration, with or without gingiva design, depending on the state of the soft tissues. This helps to provide a smile design and offer the clinician and patient a predictable outcome in relation to the possibilities available for the next design stages. This first digital wax-up should take into account the desired tooth position and occlusal relationship, while reaching all implant connections. The first design serves as a validation tool, using a 3D-printed restoration for the functional and esthetic evaluation. Following the approval of the design project by the clinician, the full-arch restoration design is then exported from Exocad (exocad GmbH, Darmstadt, Germany) and imported into Blenderfordental (Blenderfordental, Gold Coast, Australia) for the sectioning of the bar. The process offers the ability to connect the implant abutments from any digital library followed by the overlapping with the final restoration. This connection between implants can be edited and shaped according to the technician and after the clinician’s requirements by having a specific margin, thickness, volume, shape and even retentions. At this stage, the technician can also edit the superstructure contour, in order to ensure that the screw holes are in the desired locations. After final editing, the software reverse engineers the bar, by cutting it from the original design and creating two independent components, without the requirement of overcontouring the final restoration, as in a classic protocol, where the bar is created before the final design (Figure 2). The software also provides the ability to select the cement layer thickness; therefore, by having a stable and tested protocol, the dental team can achieve an optimal fit.

#### 4.2.3. Manufacturing and Delivery

**Figure 3:**
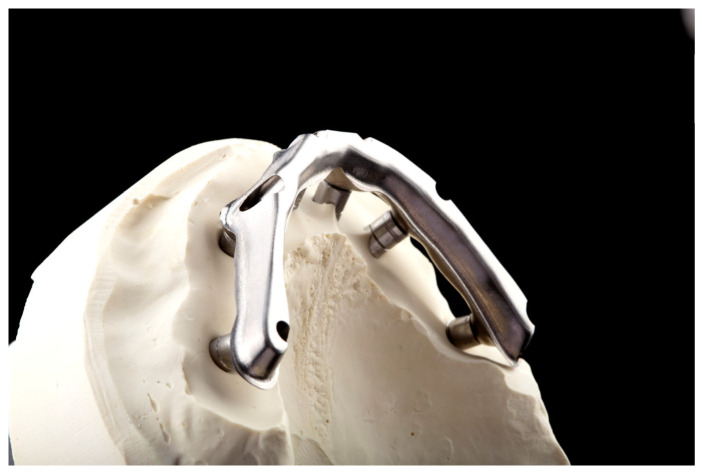
Milled bar on working model.

**Figure 4:**
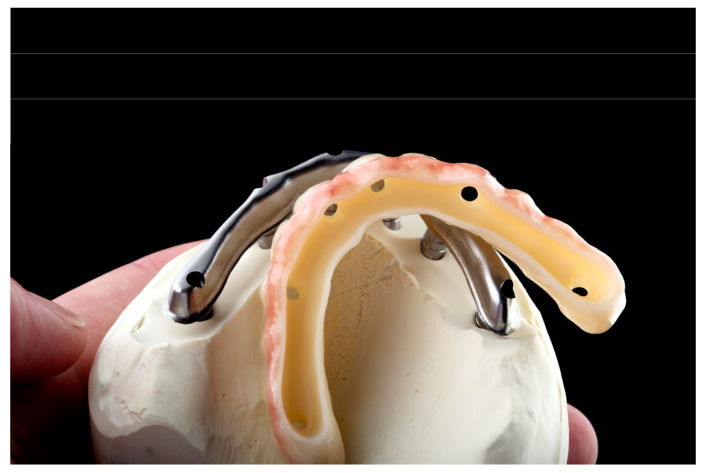
PMMA try-in used for fit validation.

**Figure 5:**
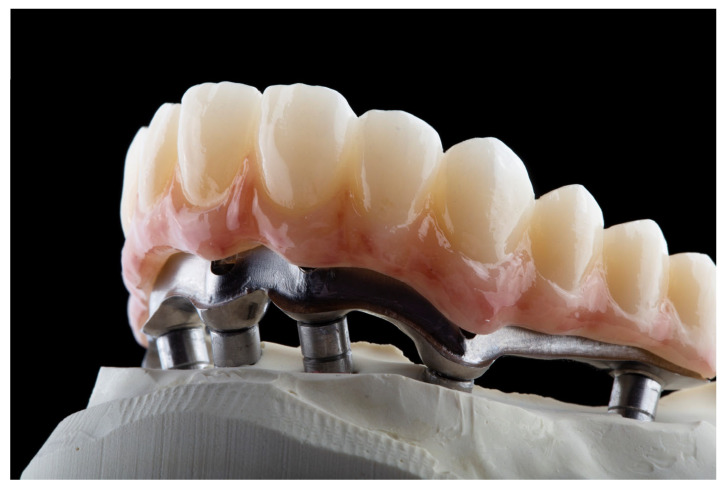
Processed zirconia ready for luting.

**Figure 6:**
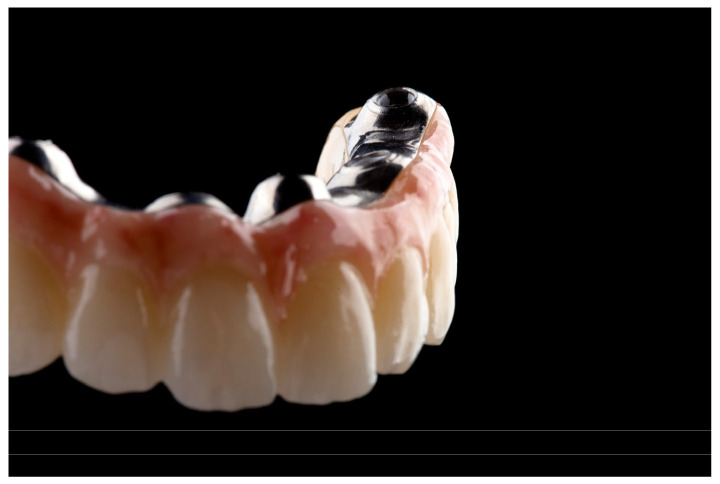
Final restoration delivered to dental office.

**Figure 7:**
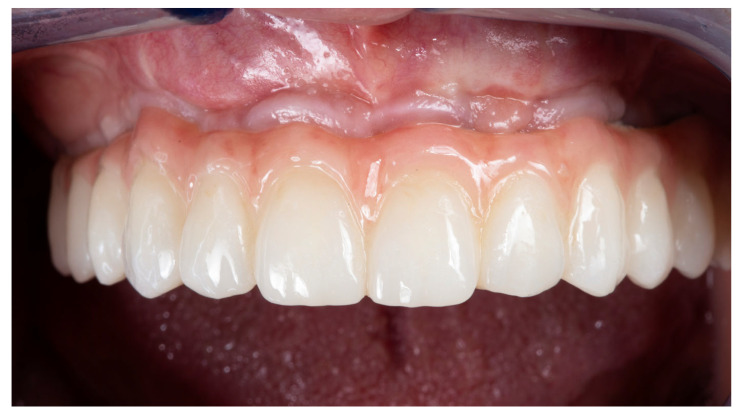
Intraoral application of restoration.

The bar (Figure 3) is milled with the multi-unit abutment connection included, although the alternative of cementing the multi-unit ti-bases intraorally may help mitigate any misfit that might occur during the protocol, with the amendment that it introduces another interface and potential risk. A PMMA try-in (Figure 4) is then milled and used for the validation of fit, in order to commence the milling of the final zirconia. Following zirconia milling, sintering, staining and glazing (Figure 5), the structures are luted together, providing the final restoration (Figure 6). Final touches can be made, in order to ensure that no marginal gap is visible, the surface is polished with no excess cement and there are no retentive spaces. The final dental prosthesis is then delivered in the office setup, where it is applied intra-orally (Figure 7).

#### 4.2.4. Follow-Up

Follow-up assessments were conducted at 1 and 6 months, during which no complications were observed regarding the implants, bar or superstructure and oral hygiene was deemed satisfactory. However, no index measurements were recorded at these intervals. The patient is scheduled for a 12-month follow-up evaluation.

## 5. Discussion

Introducing a novel technique with limited studies requires a thorough consideration of its advantages and considerations to properly evaluate its efficacy as a restoration method.

### 5.1. Passivity and Accuracy

Passivity remains a critical factor influencing the clinician’s choice of materials and production method for restorations, particularly concerning their requirements of dental technicians. Studies dating back to 1991 suggest that misfits exceeding 150 microns increase the risk of complications [52,53]. Traditional casting techniques, with their multiple steps, may introduce human errors and reduce precision and predictability, prompting a shift towards CAD/CAM and CNC technologies, known for providing superior fit [28]. Although non-passive frameworks can be corrected through sectioning and welding, this adds complexity and potential errors. Novel approaches use milled titanium and zirconia as primary materials aiming to maintain passivity, which are crucial for ensuring both the longevity of the restoration and the health of the underlying implants. The use of intraoral scanners has proven to be comparable to the conventional impression [54] and the fabrication process through milling provides great consistency and low deviation, with the fit reported being in the range of 3.91–6.91 μm for titanium and 3.89–7.8 μm for Co-Cr alloy for 3-unit FDPs [55]. The current proposed restoration type takes into account the benefits of milled titanium as an interface towards the implants, as full-arch zirconia fixed prostheses have been reported to tolerate less stress [56]. Another possibility would be the use of custom implant components, such as OT Equator components, in order to mitigate the potential lack of passivity [49]. A study from 2015 shows that zirconia frameworks have a potential error as high as 103.81 ± 43.15 µm in a three-implant setup similar to Brånemark Novum, while milled titanium, for a five-implant setup, has shown total errors of 37 ± 18 µm as early as 1999 [57,58]. This difference should be of great significance when choosing between full-arch zirconia and using a titanium bar, as passivity issues can have great detrimental effects on overall treatment.

### 5.2. Strength and Flexibility

Both titanium and zirconia boast satisfactory mechanical properties, making them favored choices for long-term restorations, while being studied for their role in full-arch fixed prosthodontics [13,46]. However, studies regarding the mechanical requirements of milled titanium bars are limited. Understanding the minimum thickness and stress behavior of such bars is crucial for successful implementation, as the proposed protocol calls for the substraction of the bar from the ideal restoration. Although parameters for milling and geometry limits are available, research is needed to evaluate the properties of zirconia-luted titanium bars and their potential for thinner designs.

Additionally, the lower modulus of elasticity of titanium may offer certain advantages over zirconia, particularly in mitigating misadaptation issues [59,60]. This would suggest the possibility of utilizing a sandwich-type restoration combining both materials for improved outcomes. However, more research is required in order to properly assess the behavior of such a restoration.

### 5.3. Weight

When designing a full-arch implant-supported fixed restoration, especially for cases with a significant volume to be restored, it is essential to consider the final weight of the restoration due to its potential impact on the underlying implants through stress distribution [61]. Ideally, a higher number of implants and a lighter restoration are preferred, necessitating materials with good mechanical properties and lower density. While full-arch zirconia offers satisfactory restoration, it comes with a large restored volume and a high density of approximately 6 g/cm^3^ [62]. The proposed alternative involves substituting some zirconia with titanium, without modifying the shape of the restoration, thereby reducing the overall weight as titanium has a lower density of 4.5 g/cm^3^ [63]. By utilizing a 3D dental design software, a study in 2020 calculated the weight of multiple restorations based on a standardized volume of 8.09 cm^3^ [61]. For instance, a full-arch full zirconia restoration is estimated to weigh approximately 48.5 g, whereas using titanium would reduce the weight by at least 25% for the substituted volume. Although using acrylic resin instead of zirconia would further reduce weight, viable aesthetic and durable options are lacking when considering a polymer for the final restoration. Moreover, should the restoration be an FP2- or FP3-type restoration, the substituted volume increases considerably, with a negative impact on the weight and therefore on the predictability of the outcome and the comfort of the patient. Although a study from 2020 showed that a 60 g restoration supported on four implants does not induce enough strain for bone remodelling [61], the possibility of having an alternative raises questions regarding the clinician’s choice for materials and technique. The software employed by the proposed protocol sections the bar from the desired outcome, which in turn reduces the required volume of the restoration. This option comes as an improvement over the design protocol that involves bar design before considering the final position of the teeth. These aspects, while benefitting weight reduction, pose the risk of reducing the thickness of components and introducing weak points in the design. Further analysis is required in order to assess minimal thickness and stress distribution in relation to the restoration geometry.

### 5.4. Biological Aspects and Interfaces

Despite zirconia and titanium’s known biocompatibility, the biological considerations here focus on the indication of a fixed restoration over a removable one. A review and meta-analysis in 2022 highlighted inconsistencies in the literature concerning the periodontal status during the follow-up period of patients treated with all-on-x-type restorations [9]. While the removable prostheses group showed a higher cumulative implant loss over five years, the prevalence of peri-implant disease varied greatly, ranging from 10% to 50% for the fixed prosthesis group [9]. Although fixed treatment options should yield better outcomes [9], the proposed technique introduces another interface between titanium and zirconia, which may influence plaque retention due to the cement layer and the potential for the development of marginal gaps, which in turn could affect peri-implant tissue health. However, the acceptable clinical ranges stated by Bhaskaran E et al. in 2013 were between 10 and 160 μm for the vertical marginal gap [64], which is higher than the predictable misfit of the proposed restoration. Therefore, the passivity of the framework may prove more relevant over time than the risk presented by adding another interface. Furthermore, the luting protocol is carried out in a dental technician’s laboratory environment, providing control over the fit and luting of the superstructure to the bar. This aspect warrants further research to better understand its implications, considering that it could be a main disadvantage, other than financial and learning requirements.

### 5.5. Maintenance

Over time, all full-arch restorations necessitate follow-up, with certain complications being more frequent than others. The proposed technique may lead to complications involving implants, framework, or esthetic components. Common reported complications are usually of a technical nature and include chipping of either the restoration or of the antagonist, material fractures, or debonding of veneering material [65]. Complications related to the framework, such as screw fracture or loosening or framework fracture, are also reported [66]. However, the digital workflow allows for the identical reproduction of any of the parts involved, allowing for the same restoration to be recreated easily. This type of restoration, along with the other options using zirconia, also brings the possibility of in-office repair with composite resin as described by Mesquita et al. [67]. While full-arch zirconia may pose greater issues when chipping because of the risk of fracture, options that use a framework may risk the fracture of the superstructure and the debonding from the bar.

### 5.6. Options

The flexibility of the proposed workflow allows for various materials to replace any component if the need arises. In cases requiring a softer material, a try-in or a provisional restoration, polymethyl methacrylate (PMMA) can substitute for zirconia while maintaining the same restoration design. The entire structure can be printed through stereolithography (SLA) technology to verify the design and make adjustments before committing to the framework or superstructure. This adaptability also permits adjustments based on the patient’s financial situation, with milled composite serving as a cost-effective alternative to zirconia. Regardless of the chosen option, patient satisfaction remains higher compared to a removable overdenture [11].

## 6. Conclusions

- Material and technology advancements:
  ○ Modern digital workflows offer clinicians a wide range of materials and techniques that encompass both analog and digital approaches for fixed dental prostheses (FDPs), each with specific strengths and limitations.
- Proposed technique combines:
  ○ Passivity, strength, and weight reduction via a titanium framework.
  ○ Aesthetic benefits and durability through a zirconia superstructure.
- Reverse engineering process:
  ○ Utilizes advanced software with a dual software approach to reverse engineer the titanium bar based on the desired restoration.
  ○ Enhances the esthetic outcome by optimizing the design for predictability, volume, and weight reduction.
- Potential limitations:
  ○ The presence of an additional interface could present long-term challenges.
  ○ Evidence supporting the long-term biological and mechanical performance of hybrid restorations, such as those combining titanium and zirconia, is currently limited.
- Future directions:
  ○ Further research is needed, including clinical follow-up and comparative studies to assess the outcomes and long-term performance of various FDP restoration options.
  ○ Comparative research should focus on evaluating analog and digital workflows to identify best practices for specific clinical scenarios.
  ○ Continued advancements in cost-effective, user-friendly digital solutions can help bridge gaps in accessibility and adoption, while maintaining a biologically driven treatment approach.

## Data Availability

Not applicable.
